# SUVN‐L3307032, A Positive Allosteric Modulator (PAM) at Muscarinic M4 Receptor for the Treatment of Neuropsychiatric Symptoms

**DOI:** 10.1002/alz.087845

**Published:** 2025-01-03

**Authors:** Anil Shinde, Naga Sai Soma Chandra Sekhar Guduru, Ankit Das, Keerthi Sai Bitra, Hima Bindu Bobbili, Kavya Sree Mukkollu, Chaitanya Sree Allu, Shivani Vanga, Radhey Shyam, Rajesh Kallepalli, Renny Abraham, Ramakrishna Nirogi

**Affiliations:** ^1^ Suven Life Sciences, Hyderabad, Telangana India

## Abstract

**Background:**

Centrally acting muscarinic acetylcholine receptor antagonists like atropine and scopolamine can induce psychosis‐like symptoms. Xanomeline, a muscarinic M1/M4 preferring agonist attenuated the effects of amphetamine (animal model for schizophrenia) in the wild‐type mice, however, such effects were absent in muscarinic M4 knockout mice. In addition, xanomeline was also found to be effective in attenuating neuropsychiatric symptoms. Thus, muscarinic M4 agents can modulate brain circuitry that is dysregulated in disease states.

**Method:**

The effects of SUVN‐L3307032 on the allosteric site of the M4 receptor were evaluated using the cell‐based reporter gene assay. The pharmacokinetic properties of SUVN‐L3307032 were studied both in rodent and non‐rodent species. SUVN‐L3307032 was assessed for its effects on amphetamine‐induced hyperlocomotion in rats at doses of 3, 10, 30, and 60 mg/kg. Preliminary toxicity studies were conducted in rats and dogs to evaluate the safety of SUVN‐L3307032.

**Result:**

SUVN‐L3307032 was found to be a positive allosteric modulator of muscarinic M4 (M4‐PAM) receptor. SUVN‐L3307032 showed good oral bioavailability in rats and dogs. It also has excellent brain penetration with an adequate free fraction in the brain. SUVN‐L3307032 showed dose‐dependent receptor occupancy at tested doses of 3, 10, 30, and 60 mg/kg. At the tested doses, SUVN‐L3307032 dose‐dependently attenuated amphetamine‐induced hyperlocomotion. The observation from the amphetamine‐induced hyperlocomotion assay correlated well with the occupancy at the allosteric site of muscarinic M4 receptors.

Preliminary toxicity studies did not show any concerns for further development.

SUVN‐L3307032 is being further characterized in animal models for neuropsychiatric symptoms.

**Conclusion:**

SUVN‐L3307032 could be a promising agent for the management of neuropsychiatric symptoms.

